# Age-Stratified Seroprevalence of SARS-CoV-2 Antibodies before and during the Vaccination Era, Japan, February 2020–March 2022

**DOI:** 10.3201/eid2811.221127

**Published:** 2022-11

**Authors:** Seiya Yamayoshi, Kiyoko Iwatsuki-Horimoto, Moe Okuda, Michiko Ujie, Atsuhiro Yasuhara, Jurika Murakami, Calvin Duong, Taiki Hamabata, Mutsumi Ito, Shiho Chiba, Ryo Kobayashi, Satoshi Takahashi, Keiko Mitamura, Masao Hagihara, Akimichi Shibata, Yoshifumi Uwamino, Naoki Hasegawa, Toshiaki Ebina, Akihiko Izumi, Hideaki Kato, Hideaki Nakajima, Norio Sugaya, Yuki Seki, Asef Iqbal, Isamu Kamimaki, Masahiko Yamazaki, Yoshihiro Kawaoka, Yuki Furuse

**Affiliations:** University of Tokyo, Tokyo, Japan (S. Yamayosh, K. Iwatsuki-Horimoto, M. Okuda, M. Ujie, A. Yasuhara, J. Murakami, C. Duong, T. Hamabata, M. Ito, Y. Kawaoka);; National Center for Global Health and Medicine, Tokyo (S. Yamayoshi, Y. Kawaoka);; University of Wisconsin–Madison, Madison, Wisconsin, USA (S. Chiba, Y. Kawaoka);; Sapporo Medical University Hospital, Sapporo, Japan (R. Kobayashi);; Sapporo Medical University School of Medicine, Sapporo (S. Takahashi);; Eiju General Hospital, Tokyo (K. Mitamura, M. Hagihara);; Japanese Red Cross Ashikaga Hospital, Ashikaga, Japan (A. Shibata);; Keio University School of Medicine, Tokyo (A. Shibata, Y. Uwamino, N. Hasegawa, N. Sugaya);; Yokohama City University Medical Center, Yokohama, Japan (T. Ebina, A. Izumi);; Yokohama City University Hospital, Yokohama (H. Kato);; Yokohama City University Graduate School of Medicine, Yokohama (H. Kato, H. Nakajima); Keiyu Hospital, Yokohama (Y. Seki);; National Hospital Organization Saitama Hospital, Wako, Japan (A. Iqbal, I. Kamimaki);; Zama Children’s Clinic, Zama, Japan (M. Yamazaki);; Nagasaki University Graduate School of Biomedical Sciences, Nagasaki, Japan (Y. Furuse);; Nagasaki University Hospital, Nagasaki, Japan (Y. Furuse)

**Keywords:** COVID-19, coronavirus disease, SARS-CoV-2, severe acute respiratory syndrome coronavirus 2, viruses, respiratory infections, zoonoses, vaccine-preventable diseases, seroprevalence, antibody, vaccine, epidemiology, incidence, Japan

## Abstract

Japan has reported a relatively small number of COVID-19 cases. Because not all infected persons receive diagnostic tests for COVID-19, the reported number must be lower than the actual number of infections. We assessed SARS-CoV-2 seroprevalence by analyzing >60,000 samples collected in Japan (Tokyo Metropolitan Area and Hokkaido Prefecture) during February 2020–March 2022. The results showed that ≈3.8% of the population had become seropositive by January 2021. The seroprevalence increased with the administration of vaccinations; however, among the elderly, seroprevalence was not as high as the vaccination rate. Among children, who were not eligible for vaccination, infection was spread during the epidemic waves caused by the SARS-CoV-2 Delta and Omicron variants. Nevertheless, seroprevalence for unvaccinated children <5 years of age was as low as 10% as of March 2022. Our study underscores the low incidence of SARS-CoV-2 infection in Japan and the effects of vaccination on immunity at the population level.

SARS-CoV-2, the etiologic agent of COVID-19, emerged at the end of 2019 and caused a pandemic. As of April 2022, despite the development of effective vaccines and therapeutics, >500 million persons had been infected with the virus and ≈6 million had died (*1*). In Japan, with a population of ≈125 million, the reported numbers are ≈7 million infections and ≈30,000 deaths by that time (*2*); however, the actual number of infected persons must be higher than the reported figure because not all infected persons undergo diagnostic testing.

A serologic survey can retrospectively find persons who have been infected with the virus (*3*). Antibodies against the SARS-CoV-2 spike protein are generated by vaccination and natural infection. In contrast, antibodies against other components of the virus, such as the nucleoprotein, represent a history of SARS-CoV-2 natural infection but not vaccination with the COVID-19 vaccines currently available in Japan. Analyses of seroprevalence in several countries have revealed that the actual incidence of SARS-CoV-2 infection is much higher than the reported COVID-19 cases (*3*). For example, in the United States, the seroprevalence of antibodies against the SARS-CoV-2 nucleoprotein ranged from 3% to 10% in 2020 (*4*–*7*), and this number reached roughly 20%–60% in 2021 (*8*,*9*). However, diagnostic tests confirmed only a fraction of the infections, especially at the beginning of the pandemic. The ascertainment rate was <10%–30% in 2020 (*4**–**7*) and increased to ≈50% in 2021 (*10*).

Vaccines for SARS-CoV-2 can prevent severe illness and death from COVID-19 for persons at high risk, such as the elderly (*11*). In addition, they can prevent viral infection and therefore have the potential to contribute to herd immunity and the containment of the disease (*12*). However, the continuous emergence of novel variants of SARS-CoV-2 and waning immunity have enabled the pandemic to linger (*13*,*14*).

We measured the seroprevalence of antibodies against the spike protein of SARS-CoV-2 in Japan by analyzing >60,000 samples obtained during February 2020–March 2022. We compared the results with the number of reported COVID-19 cases to discuss the actual incidence and the ascertainment rate. Furthermore, our findings reveal how vaccination influenced COVID-19 immunity in Japan at the population level.

## Methods

### Study Participants and Samples

The study participants were patients who visited Sapporo Medical University Hospital, Japanese Red Cross Ashikaga Hospital, Keio University Hospital, National Hospital Organization Saitama Hospital, Eiju General Hospital, Yokohama City University Medical Center, Yokohama City University Hospital, Keiyu Hospital, or Zama Children’s Clinic, Japan, during February 2020–March 2022. Sapporo Medical University Hospital is located in Hokkaido Prefecture, whereas all of the other healthcare facilities are located in the Tokyo Metropolitan Area and its suburbs.

We analyzed residual serum or plasma samples collected for medical examination. The reason for the healthcare facility visit was not considered for inclusion in this study, except that patients positive by SARS-CoV-2 nucleic acid test or antigen test were excluded. Because the samples were collected anonymously, some of them might have been from multiple visits by the same patients, but we could not identify or exclude them.

### Measurement of Antibodies

We performed ELISA to detect antibodies against SARS-CoV-2 as described previously (*15*). We incubated 96-well MaxiSorp microplates (ThermoFisher, https://www.thermofisher.com) with 2 μg/mL of the recombinant receptor-binding domain (RBD) of the spike protein, the whole length of the nucleoprotein, or phosphate-buffered saline (PBS) at 4°C overnight. We then incubated the microplates with 5% skim milk in PBS containing 0.05% tween-20. We incubated the antigen-coated microplates with the serum or plasma samples 40-fold diluted in 5% skim milk in PBS containing 0.05% tween-20, followed by the peroxidase-conjugated goat antihuman IgG, Fcγ fragment–specific antibody (Jackson ImmunoResearch Laboratories, https://www.jacksonimmuno.com). We added One-Step Ultra TMB-Blotting Solution (ThermoFisher) to each well and incubated for 3 min at room temperature. We stopped the reaction by adding 2 M H_2_SO_4_ and immediately measured the optical density at 450 nm (OD_450_). We subtracted the OD_450_ value of the PBS wells from the OD_450_ value of the spike protein or nucleoprotein wells as background.

### Validation Samples for ELISA

We used convalescent serum samples from patients with laboratory-confirmed COVID-19 as positive controls to validate the ELISA tests. We used residual serum samples collected in 2012 as negative controls.

### Other Data Sources

We obtained the daily number of reported COVID-19 cases from the website and press releases of each prefecture in the study area. Confirmation and reports of COVID-19 were based on PCR testing at the beginning of the pandemic, and antigen testing, which was approved and used after May 2020. Vaccine administration data were available in the Vaccination Record System (https://cio.go.jp/vrs). This system was launched in April 2021, and the number of vaccines administered before that timepoint was included on the first day of the record. All vaccines available in Japan require 2 doses for immunization; a third dose was administered as a booster after December 2021. We downloaded and used census data of Japan to obtain demographic information in the study area (https://www.stat.go.jp/data/jinsui/2021np/index.html).

### Statistical Analysis

We drew a receiver operating characteristic curve for the ELISA OD_450_ values to set a threshold. We used this threshold to determine whether samples were negative or positive for SARS-CoV-2 spike protein and nucleoprotein by using Youden’s index (*16*).

We investigated the proportion of seropositive samples by month and age group. We computed the Wilson 95% CI for the seroprevalence data (*17*). Using the census data, we calculated an age-structure adjusted estimation of seroprevalence in the total population and the rates of reported COVID-19 cases and vaccine administrations.

### Ethics Considerations

The study protocol was reviewed and approved by the institutional review board of the Institute of Medical Science, University of Tokyo (protocol no. 2019-75). The protocol was also checked and approved by each research institute and healthcare facility involved. The study participants gave informed consent during their healthcare facility visits for their data and residual samples to be used anonymously for clinical research.

## Results

**Figure 1:**
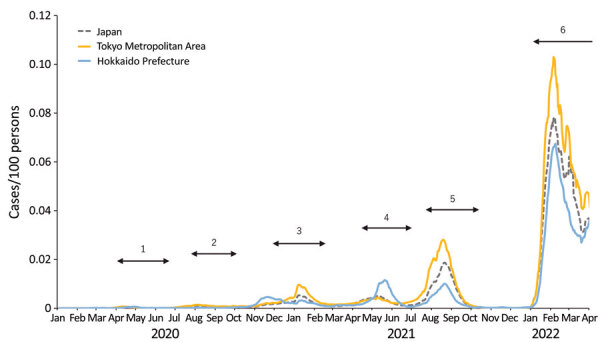
Epidemic curve of COVID-19 in Japan, January 2020–March 2022. The daily numbers of reported COVID-19 cases per100 persons in all of Japan, the Tokyo Metropolitan Area, and Hokkaido Prefecture are shown. The numbers indicate the 6 epidemic waves. The fourth, fifth, and sixth waves were driven by the Alpha, Delta, and Omicron variants of SARS-CoV-2, respectively.

During the study period, Japan had 6 COVID-19 epidemic waves (Figure 1). The cumulative number of confirmed COVID-19 cases by the end of March 2022 was ≈6.7 million in a population of ≈125 million. Vaccinations started in February 2021 for healthcare workers; then, in April 2021, they were expanded to the general population, prioritizing persons at high risk, such as the elderly and those with certain underlying conditions, including respiratory disorders and immunocompromised diseases. Approximately 256.9 million doses of vaccine were administered during the study period. The 2 mRNA vaccines, BNT162b2 (Pfizer-BioNTech, https://www.pfizer.com) and mRNA-1273 (Moderna, https://www.modernatx.com), were the main vaccines administered in Japan.

We first assessed SARS-CoV-2 antibody titers in prepandemic samples from 2012 (n = 200) and in COVID-19 convalescent serum samples (n = 113). The median time from PCR-positive result to sample collection for the serologic assay for the convalescent serum samples was 40 days (interquartile range 32–64 days). We determined the thresholds for discriminating infected convalescent samples from uninfected prepandemic samples by using receiver operating characteristic curves (Appendix Figure 1). The ELISA test for antinucleoprotein antibodies had 98.0% specificity and 95.6% sensitivity.

The antibody titer for the RBD of the spike protein, which has a specificity of 99.5% and a sensitivity of 100%, can be used to clearly differentiate convalescent samples from naive samples. Hence, we measured the antibody titers for the spike protein in further analyses. Our seroprevalence data cannot determine whether immunity was generated by natural infection or vaccination.

We also checked whether our assay could detect the history of infection with SARS-CoV-2 variants, such as Delta and Omicron. We ensured that the sensitivity of the assay for the anti–spike protein antibodies did not decrease because of antigenic changes in such variants, confirming 100% positivity in samples from unvaccinated persons infected with those variants (24/24 for Delta and 5/5 for Omicron).

We collected a total of 44,681 samples in the Tokyo Metropolitan Area during February 2020–March 2022. Of these samples, 44,672 (99.9%) were analyzed for the study, and 9 were excluded because the metadata were incomplete. We collected the samples from persons 0 to 105 years of age and summarized the numbers of analyzed samples by age group and month (Appendix Table 1).

**Figure 2:**
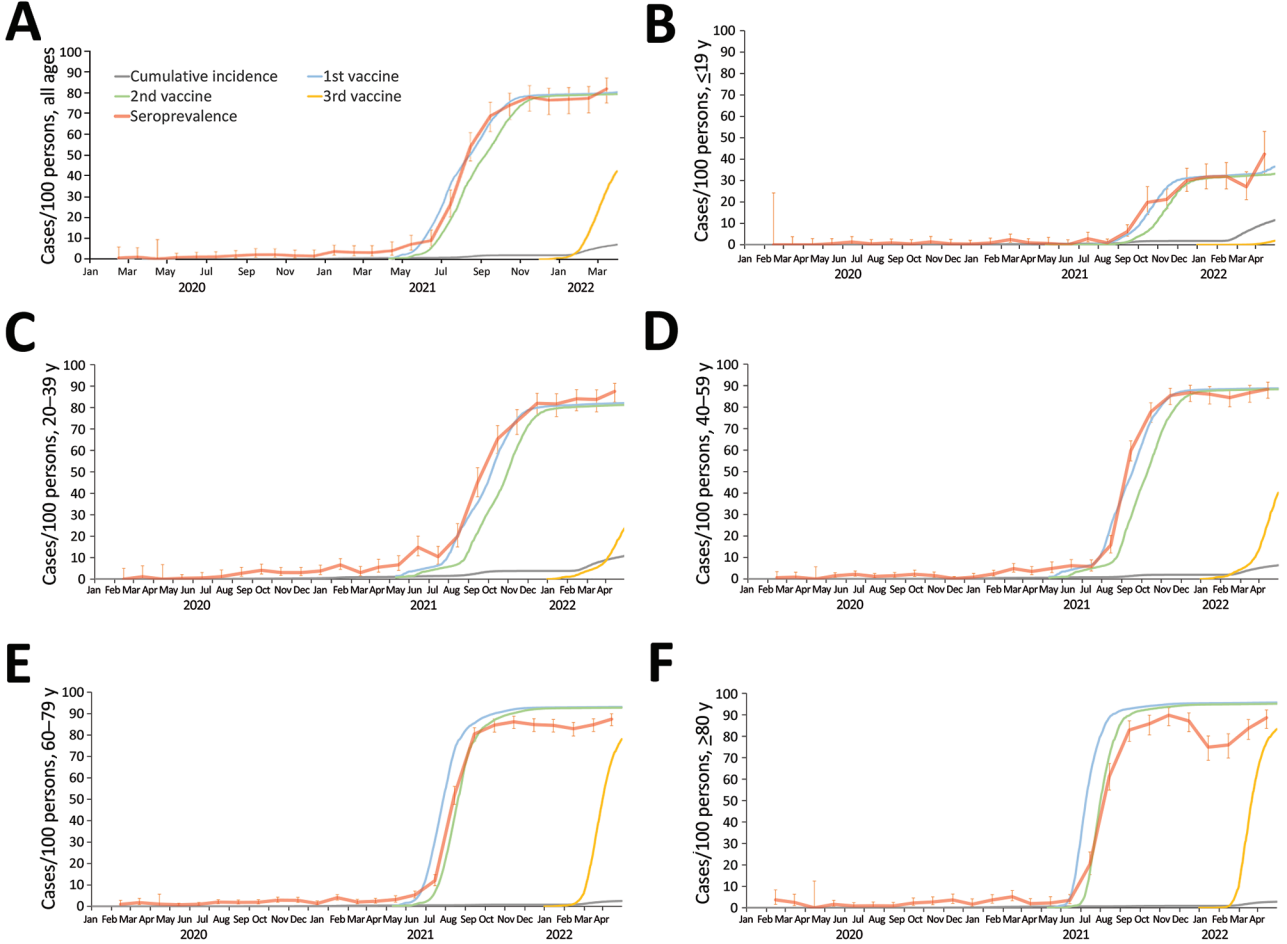
Seroprevalence of SARS-CoV-2 in the Tokyo Metropolitan Area, Japan, February 2020–March 2022. A) Rates for the total population of the Tokyo Metropolitan Area; B–F) rates by 20-year age groups. The cumulative number of reported COVID-19 cases and the cumulative number for the first, second, and third vaccine administrations per population are also shown. Error bars indicate 95% CIs. Detailed age-stratified data are shown in Appendix Figure 2).

SARS-CoV-2 seroprevalence was low in 2020 in the Tokyo Metropolitan Area (Figure 2; Appendix Figure 2). In January 2021, just before the vaccine rollout, the estimated seropositivity was 3.8% in the total population when we adjusted our data to age structure in the area. The proportions of serum samples positive for SARS-CoV-2 in each age group at the time were 0% among persons 0–9 years of age, 2.5% among persons 10–19 years of age, 8.2% among persons 20–29 years of age, 5.7% among persons 30–39 years of age, 2.8% among persons 40–49 years of age, 2.0% among persons 50–59 years of age, 4.2% among persons 60–69 years of age, 4.0% among persons 70–79 years of age, and 3.7% among persons >80 years of age (Appendix Figure 2).

**Figure 3:**
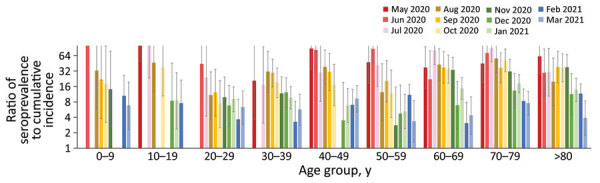
Ratios of SARS-CoV-2 seroprevalence to cumulative incidence by month in the Tokyo Metropolitan Area, Japan, May 2020–March 2021. Ratios for each month were calculated in comparison to the cumulative incidence of reported COVID-19 from January 2020 to that month. The ratio corresponds to the actual number of infected persons per reported case-patient. Error bars indicate 95% CIs. Data are blank for months when no samples were positive for SARS-CoV-2.

We then calculated the ratio of the seroprevalence to the cumulative incidence by the time of vaccination for the general public (Figure 3). This rate can correspond to the number of actual infected persons per detected case. However, a low antibody titer in some infected persons because of a weak immune response and waning immunity could affect the accuracy of the estimation.

In the early phase of the pandemic, diagnostic tests detected as few as 1 case in >10 infections (an ascertainment rate of <10%). The rate increased over time, such that by March 2021, one case in ≈3–10 infections was detected (an ascertainment rate of ≈10%–33%). This change is probably attributable to an increase in the proportion of infected persons who underwent diagnostic testing rather than an improvement in testing accuracy.

The proportion of samples positive for antibodies against the SARS-CoV-2 spike protein dramatically increased with the rollout of vaccination (i.e., after April 2021) (Figure 2). However, the seropositive proportions were slightly lower than the vaccination rates in persons 70–79 and >80 years of age (Appendix Figure 2). Furthermore, seropositive rates peaked and then declined for persons >50 years of age. The administration of the third vaccination after January 2022 restored the drop in seroprevalence.

The SARS-CoV-2 seroprevalence among persons 0–9 years of age increased during the Delta-dominant fifth epidemic wave, which started in July 2021, and the Omicron-dominant sixth epidemic wave, which started in January 2022. Vaccination of children >5 years of age was approved and administered after February 2022 in Japan. Therefore, we subdivided the data for the 0–9 years age group into 0–5 months, 6 months–4 years, and 5–9 years of age (Appendix Figure 2, panel C, D). The first subset age group (0–5 months of age) showed a very high seroprevalence compared to the other 2 subset age groups. The seroprevalence for the 2 older groups was low but increased after August 2021, reaching 8.0% for the 6 months–4 years age group and 9.3% for the 5–9 years age group in December 2021. In March 2022, a further increase of seroprevalence was observed in children 5–9 and 10–19 years of age.

We also tested samples from Hokkaido Prefecture, which is situated ≈800 km north of Tokyo. We collected and analyzed a total of 17,079 serum samples from Hokkaido Prefecture (Appendix Table 2). The results were comparable to those obtained from the Tokyo Metropolitan Area (Appendix Figure 3). The seroprevalence was <5% for all age groups until the vaccination program began. The seroprevalence increased as the vaccines were administered, although the older age groups showed lower seropositivity rates compared with their vaccination rates.

## Discussion

We examined the time course of seroprevalence of antibodies against SARS-CoV-2 by age group by analyzing >60,000 samples from Japan over a 25-month period. In addition to previous studies (*18*,*19*), our study expands knowledge about SARS-CoV-2 seroprevalence in the country. Diagnostic testing to identify persons infected with SARS-CoV-2 is important for gaining a better understanding of the epidemiologic situation of COVID-19. The incidence and mortality rates of COVID-19 are considerably low in Japan (*20*). However, the low number of tests per population may have caused many cases of infection to go undetected and the reported statistics may not have reflected the actual situation (*21*).

Our data show that ≈5% of the population of Japan had become seropositive for SARS-CoV-2 by January 2021. That figure is much higher than the reported number of COVID-19 cases. Still, the low rate was in stark contrast to other countries, many of which had seroprevalences >30% at that time (*3*). Nonpharmaceutical interventions, such as physical distancing and wearing a face mask, played a critical role in controlling the COVID-19 pandemic, especially in the prevaccination era. Although Japan did not impose a lockdown, the country issued a state of emergency, asking persons to stay at home and limit mass gatherings and asking businesses, including restaurants and bars, to reduce their hours or close when COVID-19 cases surged (*22*). The country also implemented a unique strategy focusing on case-clusters (*23*,*24*).

Because we measured antibodies for the viral spike protein, we could not differentiate immunity by natural infection from immunity by vaccination after February 2021. The seroprevalence among children who were not yet eligible for vaccination in December 2021 was still as low as 10% in Japan. Thereafter, the infection was spread among children during the Omicron-dominant sixth epidemic wave (*25*), and their seropositive rates gradually increased at the beginning of 2022. The especially high seroprevalence among children 0–5 months of age after August 2021 must be the result of antibodies transferred from vaccinated mothers (Appendix Figure 2, panel C, D).

Japan has achieved a high rate of SARS-CoV-2 seroprevalence among adults because of vaccinations since April 2021. A low seroconversion rate by vaccination and rapid immunity waning in the elderly have been reported at the person level (*26*,*27*). In our study, we observed this effect at the population level. In addition to vaccinating the elderly, who are at a high risk for experiencing severe illness, reducing their exposure to the virus should be key to protecting this vulnerable population. Booster shots also helped provide a high degree of population immunity.

In this study, we measured the antibody titers for the RBD of SARS-CoV-2 spike protein. Therefore, samples from both infected persons and from vaccinated persons showed positive results. Although the measurement of the antibody titers for the nucleoprotein can reflect only a history of natural infection with SARS-CoV-2, in our study, the sensitivity and specificity were not as high as the test for the spike protein (Appendix Figure 1). The low sensitivity might have been attributable to the weak immunogenicity of the nucleoprotein, and the low specificity may be attributable to cross-reactivity between the seasonal coronavirus and SARS-CoV-2. Still, we must pursue analyzing the actual infection rate, especially after vaccination rollout, by investigating the prevalence of antinucleoprotein antibodies. We should establish an assay that detects antibodies for the SARS-CoV-2 nucleoprotein without any cross-reactivity with other antigens in the future.

By testing antibodies for the spike protein, we gauged the actual incidence of COVID-19 in a prevaccine era. We validated the considerably high sensitivity and specificity of the test. Still, the estimate cannot be 100% accurate. Because of the effect of waning immunity, investigation of seropositivity could lead to an underestimation of the infection rate. In addition, our test participants may not represent the general public in Japan. Our samples were from patients who visited healthcare facilities for various reasons other than COVID-19. Persons with underlying diseases could be more cautious about healthcare issues and avoid high-risk behavior, or patients with some symptoms could have had a high pretest probability of past infection with SARS-CoV-2.

Our study highlights the very low SARS-CoV-2 infection rate in Japan. It also unveils a hurdle to maintaining a high degree of population immunity among the elderly. In future studies, we should investigate how population immunity has affected and will affect the course of the pandemic. We must explore the levels of immunity required to prevent infection, hospitalization, and death from different SARS-CoV-2 variants. Because our findings suggests that most populations in Japan have not yet been infected with the virus, the country’s current and future paths regarding the COVID-19 pandemic may continue to hold the world’s attention.

Appendix Additional information about age-stratified seroprevalence of SARS-CoV-2 antibodies before and during the vaccination era, Japan, February 2020–March 2022.
