# Supplementary material for: Age-Stratified Seroprevalence of SARS-CoV-2 Antibodies before and during the Vaccination Era, Japan, February 2020–March 2022
Source: Emerg Infect Dis. 2022 Nov;28(11):2198–205. doi: 10.3201/eid2811.221127 (PMC9622230; doi:10.3201/eid2811.221127)
Supplement: Appendix — Additional information about age-stratified seroprevalence of SARS-CoV-2 antibodies before and during the vaccination era, Japan, February 2020–March 2022. [file 22-1127-Techapp-s1.pdf]

Article DOI: https://doi.org/10.3201/eid2811.221127

# Age-Stratified Seroprevalence of SARS-CoV-2 Antibodies before and during the Vaccination Era, Japan, February 2020–March 2022

## Appendix

**Appendix Table 1.** Number of samples analyzed in the Tokyo Metropolitan Area

| | Age group, y | | | | | | | | | |
|---|---|---|---|---|---|---|---|---|---|---|
| Year–month | 0–9 | 10–19 | 20–29 | 30–39 | 40–49 | 50–59 | 60–69 | 70–79 | ≥80 | Total |
| 2020–02 | 6 | 6 | 32 | 41 | 67 | 97 | 120 | 182 | 135 | 686 |
| 2020–03 | 65 | 32 | 38 | 49 | 92 | 136 | 141 | 199 | 158 | 910 |
| 2020–04 | 79 | 48 | 20 | 33 | 30 | 34 | 50 | 45 | 27 | 366 |
| 2020–05 | 121 | 71 | 95 | 170 | 193 | 337 | 491 | 635 | 371 | 2484 |
| 2020–06 | 137 | 87 | 63 | 118 | 244 | 350 | 397 | 592 | 362 | 2350 |
| 2020–07 | 156 | 73 | 54 | 114 | 204 | 303 | 414 | 531 | 320 | 2169 |
| 2020–08 | 167 | 155 | 144 | 113 | 240 | 278 | 364 | 492 | 347 | 2300 |
| 2020–09 | 153 | 79 | 97 | 197 | 175 | 234 | 294 | 478 | 312 | 2019 |
| 2020–10 | 140 | 82 | 136 | 221 | 196 | 243 | 324 | 461 | 332 | 2135 |
| 2020–11 | 146 | 74 | 111 | 220 | 201 | 261 | 320 | 492 | 301 | 2126 |
| 2020–12 | 176 | 85 | 118 | 199 | 129 | 207 | 280 | 378 | 242 | 1814 |
| 2021–01 | 148 | 81 | 134 | 244 | 213 | 298 | 358 | 554 | 355 | 2385 |
| 2021–02 | 181 | 103 | 99 | 164 | 186 | 268 | 327 | 457 | 348 | 2133 |
| 2021–03 | 188 | 117 | 80 | 155 | 186 | 216 | 265 | 480 | 302 | 1989 |
| 2021–04 | 106 | 52 | 77 | 133 | 135 | 170 | 235 | 328 | 221 | 1457 |
| 2021–05 | 108 | 52 | 84 | 145 | 162 | 241 | 289 | 458 | 313 | 1852 |
| 2021–06 | 179 | 64 | 75 | 136 | 131 | 181 | 235 | 396 | 222 | 1619 |
| 2021–07 | 243 | 80 | 75 | 131 | 123 | 181 | 223 | 313 | 235 | 1604 |
| 2021–08 | 217 | 106 | 70 | 134 | 169 | 253 | 268 | 347 | 240 | 1804 |
| 2021–09 | 96 | 45 | 76 | 129 | 137 | 183 | 213 | 328 | 219 | 1426 |
| 2021–10 | 231 | 80 | 80 | 140 | 149 | 186 | 235 | 315 | 206 | 1622 |
| 2021–11 | 206 | 67 | 76 | 129 | 123 | 188 | 226 | 317 | 225 | 1557 |
| 2021–12 | 177 | 66 | 74 | 128 | 116 | 163 | 247 | 342 | 223 | 1536 |
| 2022–01 | 160 | 59 | 76 | 137 | 147 | 187 | 206 | 322 | 216 | 1510 |
| 2022–02 | 128 | 49 | 87 | 110 | 118 | 175 | 233 | 322 | 245 | 1467 |
| 2022–03 | 53 | 32 | 89 | 135 | 120 | 164 | 206 | 342 | 211 | 1352 |
| Total | 3767 | 1845 | 2160 | 3625 | 3986 | 5534 | 6961 | 10106 | 6688 | 44672 |

**Appendix Table 2.** Number of samples analyzed in Hokkaido Prefecture

| | Age group, y | | | | | | | | | |
|---|---|---|---|---|---|---|---|---|---|---|
| Year–month | 0–9 | 10–19 | 20–29 | 30–39 | 40–49 | 50–59 | 60–69 | 70–79 | ≥80 | Total |
| 2020–02 | 34 | 28 | 28 | 65 | 79 | 58 | 79 | 82 | 53 | 506 |
| 2020–03 | 72 | 140 | 155 | 213 | 361 | 214 | 288 | 285 | 121 | 1849 |
| 2020–04 | 21 | 22 | 64 | 147 | 155 | 116 | 113 | 120 | 68 | 826 |
| 2020–05 | 20 | 30 | 56 | 130 | 181 | 89 | 116 | 137 | 58 | 817 |
| 2020–06 | 14 | 49 | 66 | 151 | 174 | 115 | 130 | 125 | 89 | 913 |
| 2020–07 | 10 | 62 | 76 | 141 | 89 | 95 | 95 | 121 | 71 | 760 |
| 2020–08 | 9 | 60 | 48 | 95 | 67 | 68 | 72 | 78 | 39 | 536 |
| 2020–09 | 15 | 50 | 88 | 98 | 93 | 57 | 71 | 88 | 38 | 598 |
| 2020–10 | 15 | 51 | 78 | 89 | 93 | 69 | 85 | 89 | 53 | 622 |
| 2020–11 | 9 | 34 | 68 | 88 | 104 | 56 | 85 | 93 | 40 | 577 |
| 2020–12 | 9 | 54 | 61 | 97 | 81 | 55 | 76 | 102 | 38 | 573 |
| 2021–01 | 6 | 57 | 62 | 108 | 82 | 67 | 94 | 89 | 36 | 601 |
| 2021–02 | 3 | 49 | 69 | 95 | 73 | 65 | 79 | 81 | 26 | 540 |
| 2021–03 | 0 | 65 | 87 | 130 | 55 | 68 | 99 | 107 | 35 | 646 |
| 2021–04 | 1 | 27 | 67 | 120 | 99 | 56 | 85 | 99 | 29 | 583 |
| 2021–05 | 0 | 32 | 54 | 85 | 100 | 58 | 82 | 97 | 47 | 555 |
| 2021–06 | 2 | 29 | 58 | 97 | 103 | 61 | 82 | 101 | 31 | 564 |
| 2021–07 | 2 | 57 | 56 | 91 | 62 | 51 | 88 | 96 | 32 | 535 |
| 2021–08 | 3 | 60 | 68 | 120 | 96 | 64 | 105 | 105 | 43 | 664 |
| 2021–09 | 1 | 29 | 63 | 106 | 88 | 58 | 75 | 84 | 45 | 549 |
| 2021–10 | 2 | 26 | 54 | 116 | 101 | 64 | 75 | 90 | 45 | 573 |
| 2021–11 | 2 | 30 | 64 | 109 | 73 | 63 | 81 | 102 | 28 | 552 |
| 2021–12 | 2 | 47 | 66 | 97 | 92 | 57 | 64 | 97 | 32 | 554 |
| 2022–01 | 1 | 29 | 48 | 97 | 81 | 69 | 79 | 69 | 33 | 506 |
| 2022–02 | 2 | 24 | 44 | 90 | 99 | 52 | 79 | 94 | 25 | 509 |
| 2022–03 | 1 | 32 | 74 | 117 | 95 | 57 | 65 | 91 | 39 | 571 |
| Total | 256 | 1173 | 1722 | 2892 | 2776 | 1902 | 2442 | 2722 | 1194 | 17079 |

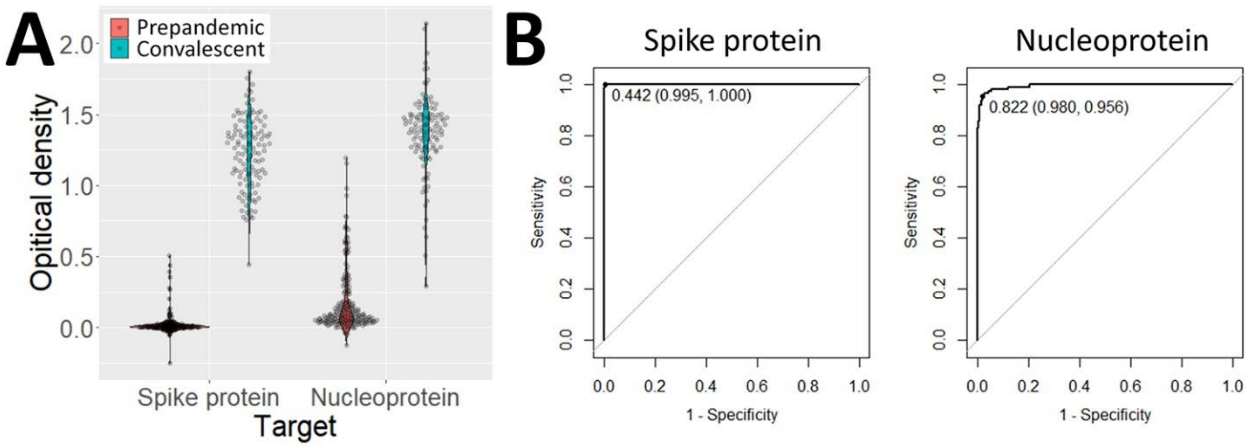


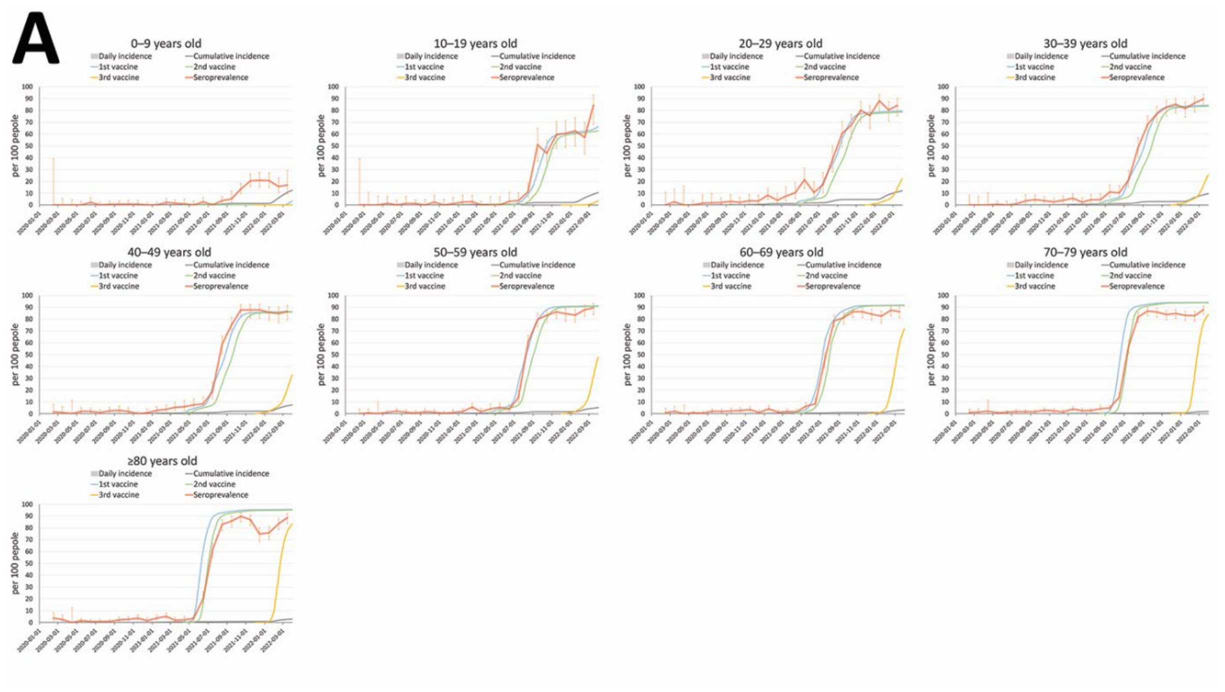


**Appendix Figure 1.** Antibody titers for SARS-CoV-2 in prepandemic and convalescent samples. A) The results of ELISA tests against the SARS-CoV-2 spike protein and nucleoprotein are shown for samples collected in 2012 (prepandemic, n = 200) and samples from COVID-19-confirmed cases (convalescent, n = 113). B) ROCs were drawn for the SARS-CoV-2 spike protein and nucleoprotein to differentiate prepandemic and convalescent samples. A cutoff value was determined by using Youden’s index. The specificity and sensitivity of the test are described in parentheses in the figure

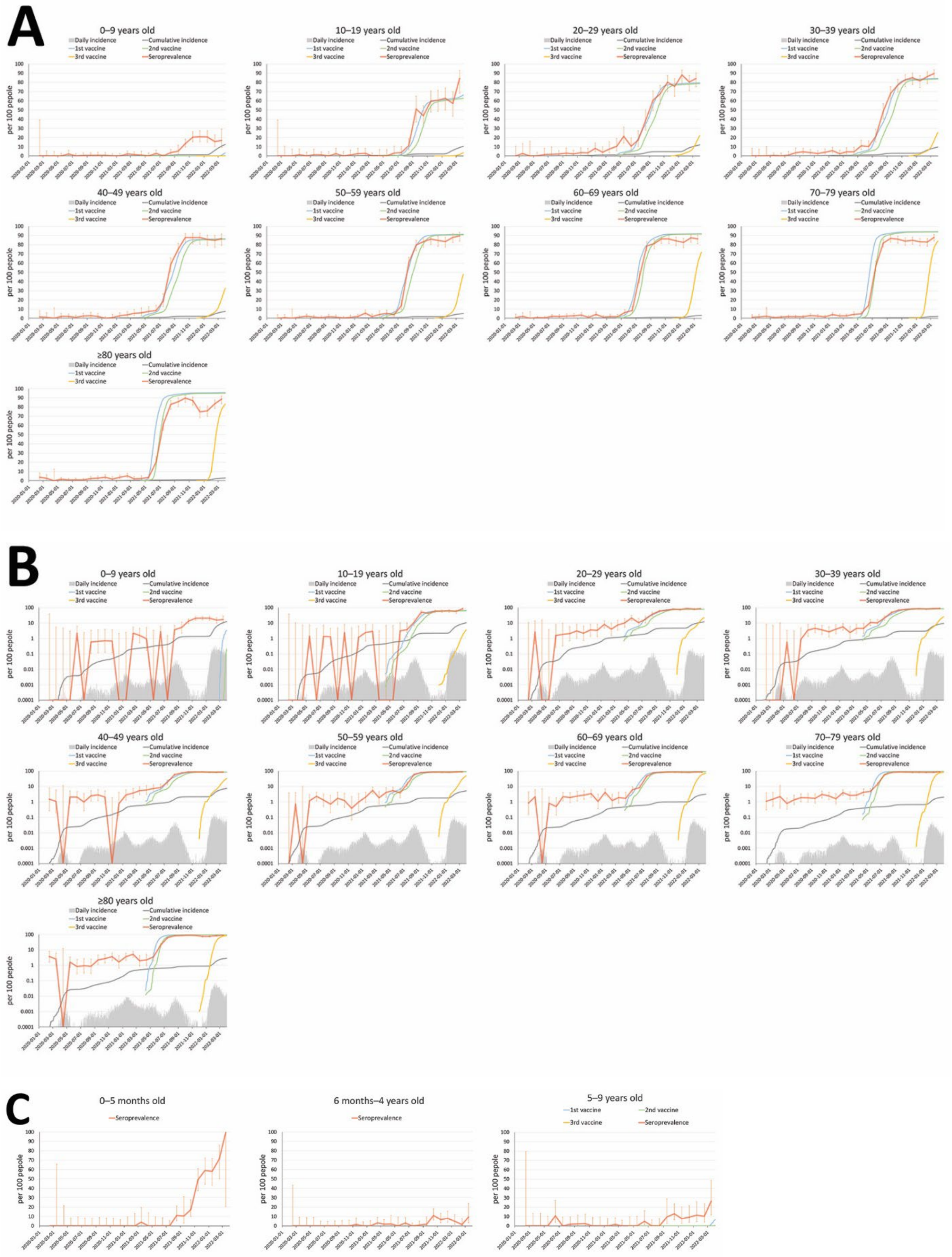
A
0–9 years old
10–19 years old
20–29 years old
30–39 years old
40–49 years old
50–59 years old
60–69 years old
70–79 years old
≥80 years old
Daily incidence
Cumulative incidence
1st vaccine
2nd vaccine
3rd vaccine
Seroprevalence
per 100 pepole
B
0–9 years old
10–19 years old
20–29 years old
30–39 years old
40–49 years old
50–59 years old
60–69 years old
70–79 years old
≥80 years old
C
0–5 months old
6 months–4 years old
5–9 years old

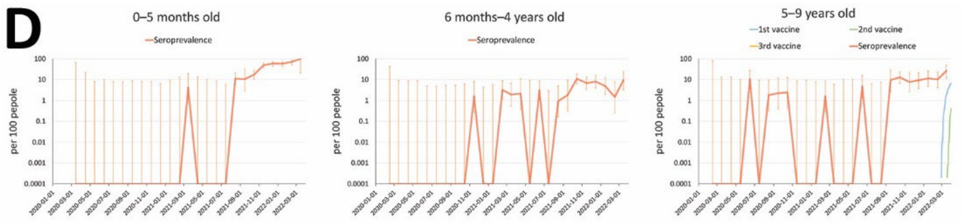


**Appendix Figure 2.** Seroprevalence of SARS-CoV-2 by age in the Tokyo Metropolitan Area, February 2020–March 2022. A) Seropositive rates for the SARS-CoV-2 spike protein in the Tokyo Metropolitan Area are shown by 10-year age group. The daily number of reported COVID-19 cases, their cumulative number, and the cumulative number for the first, second, and third vaccine administrations per population are also shown. The vertical orange lines indicate the 95% CIs of the seroprevalence data. B) The same data on a logarithmic scale are shown. C) and D) The proportions of seropositive samples in children aged 0–6 months, 6 months–4 years, and 5–9 years are shown on linear (C) and logarithmic (D) scales.

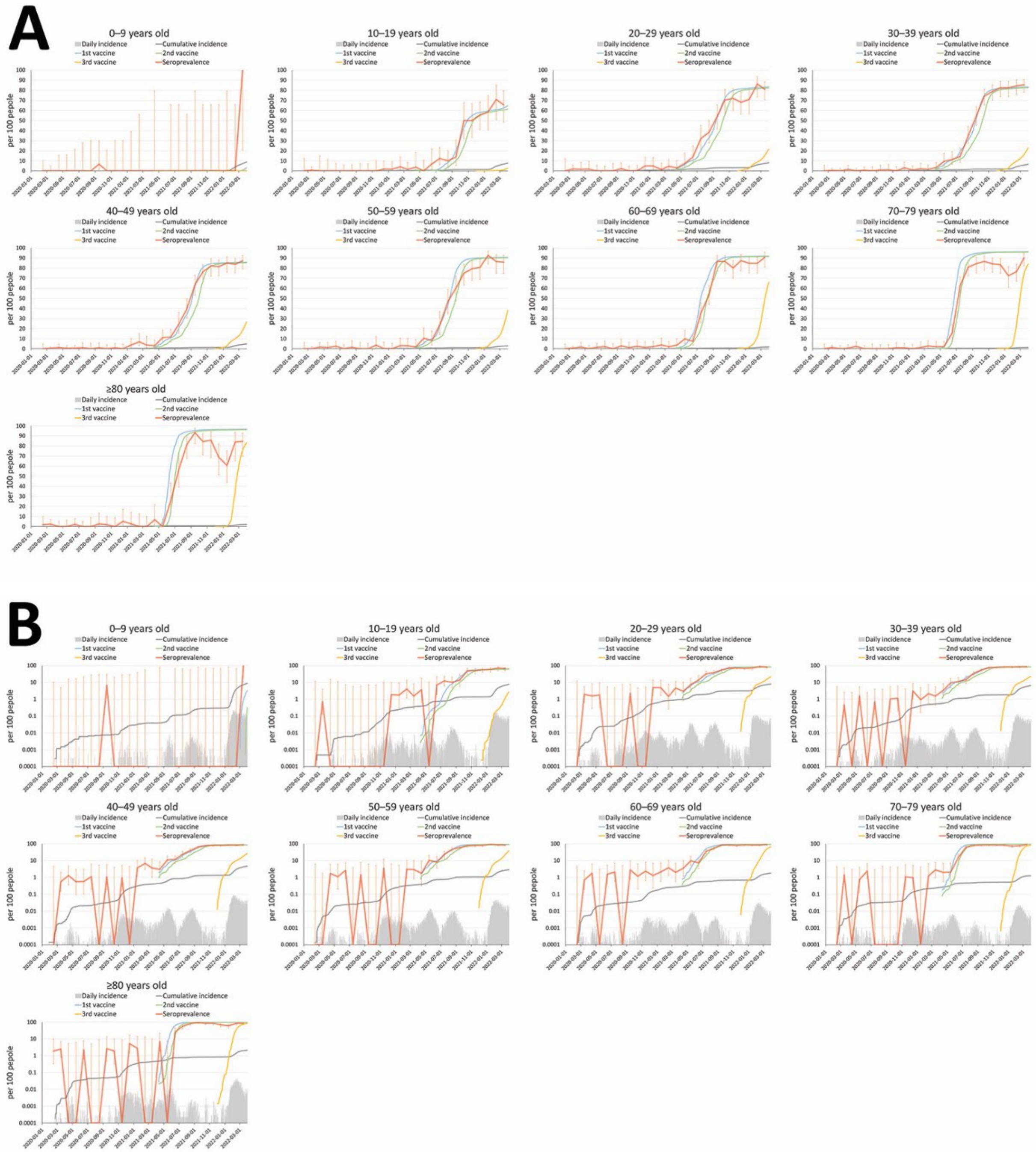


**Appendix Figure 3.** Seroprevalence of SARS-CoV-2 by age in Hokkaido Prefecture, February 2020–March 2022. A) Seropositive rates for the SARS-CoV-2 spike protein in Hokkaido Prefecture are shown by 10-year age group. The daily number of reported COVID-19 cases, their cumulative number, and the cumulative number for the first, second, and third vaccine administrations per population are also shown. The vertical orange lines indicate the 95% CIs of the seroprevalence data. B) The same data on a logarithmic scale are shown.
